# Bardet–Biedl syndrome: Delayed diagnosis in a 14‐year‐old child with end‐stage renal disease

**DOI:** 10.1002/ccr3.7649

**Published:** 2023-07-04

**Authors:** Mohammad Rasel, Ashif Istiak, Afra Saiara, Abdullah Al‐Jubair, Shariful Matin, Gobinda Chandra Roy

**Affiliations:** ^1^ Shaheed Suhrawardy Medical College Hospital Dhaka Bangladesh; ^2^ Bangladesh College of Physicians and Surgeons Dhaka Bangladesh

**Keywords:** Bardet–Biedl syndrome, central obesity, end‐stage renal disease, polydactyly, retinitis pigmentosa

## Abstract

Bardet–Biedl syndrome (BBS) is a rare autosomal recessive ciliopathic disorder. Because of its low prevalence and wide spectrum of clinical features, many patients remain undiagnosed. We report a case of a 14‐year‐old boy with a typical phenotype of BBS who remains undiagnosed until the development of end‐stage renal disease.

## Graphical abstract

A child with Bardet‐Biedl syndrome having postaxial polydactyly in both hands, micropenis, and absence of secondary sexual characteristics.

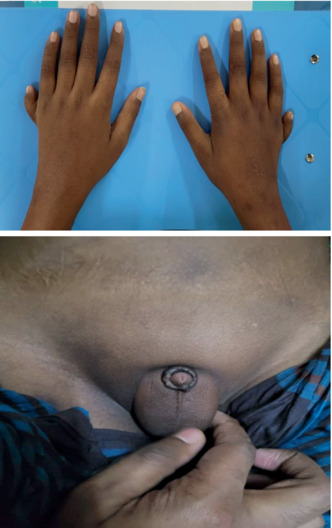

## 1 INTRODUCTION

Bardet‐Biedl syndrome (BBS) is a rare autosomal recessive disorder characterized by external deformities of limbs, obesity, visual problems, intellectual disability, and anomalies with the renal and reproductive systems.^1^ Other symptoms may also present, such as speech impediments, dental anomalies, loss of sense of smell, congenital heart disease, diabetes mellitus, liver fibrosis, etc.^1^ The most frequent cause of mortality in this syndrome is renal failure.^2^ BBS has a prevalence of 1:140000 to 1:160000 in North America and Europe, although a higher incidence has been reported in some isolated populations, such as the Bedouins of Kuwait [1:17500], people of Newfoundland [1:13500], etc.^3^ In Bangladesh, no definitive data are available regarding the prevalence of BBS. Due to its low prevalence and the wide range of symptoms, many patients remain undiagnosed.^4^ This paper reports a case of a 14‐year‐old boy who exhibited typical symptoms of BBS but was only diagnosed when he developed end‐stage renal disease.

## 2 CASE PRESENTATION

A 14‐year‐old boy was admitted into our medicine inpatient department with complaints of altered level of consciousness, scanty micturition, and respiratory distress for 2 days.

An initial evaluation revealed ESRD (End Stage Renal Disease), uremic encephalopathy, and hypertension (grade 2).

His background medical record included extra fingers in both hands, central obesity, underdeveloped genitalia with absence of secondary sexual characteristics. According to his parents, he had delayed developmental milestones with the commencement of walking and speech at ages 3 and 4 years respectively. Later, he was enrolled in a school but could not keep pace with his year mates due to his learning difficulties and poor class performances. To note, he was the first issue of his parents without consanguinity, and he was born by normal vaginal delivery at full term. There is no history of any disease running in his family. All the family members, including his only younger sister, are in good health.

**FIGURE 1:**
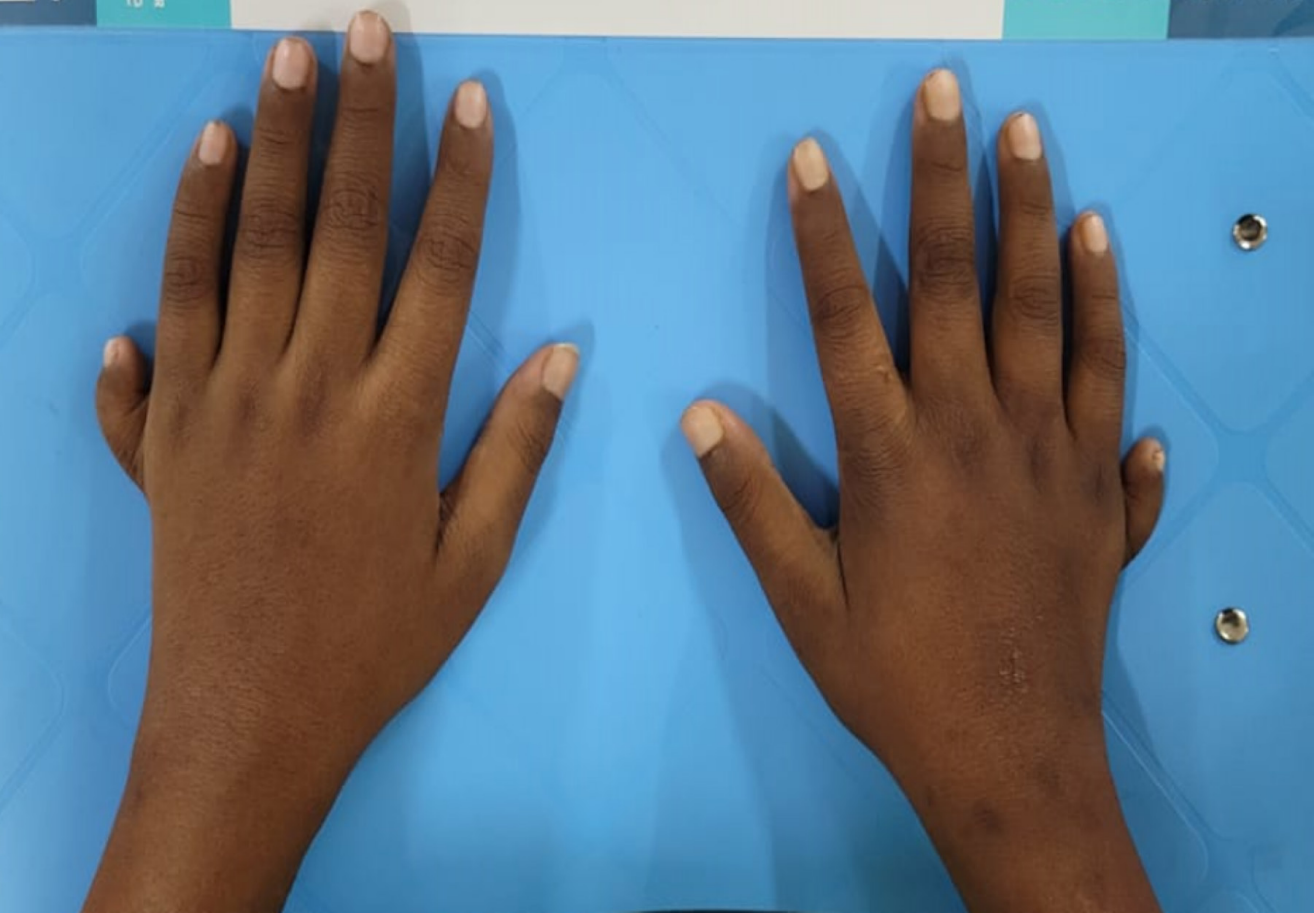
Picture showing postaxial polydactyly in both hands.

**FIGURE 2:**
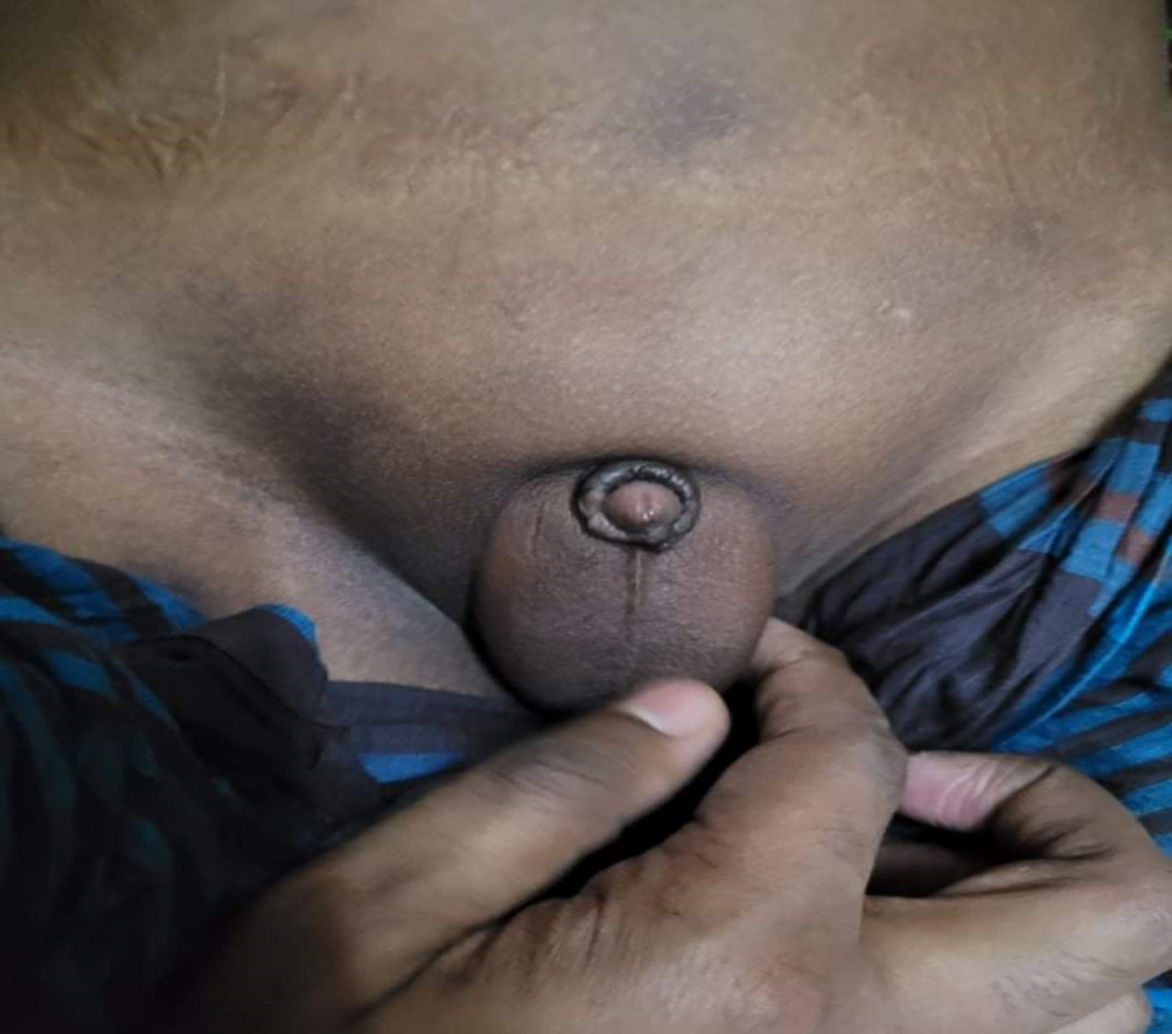
Picture showing micropenis.

On clinical examination, the boy was dyspneic, respiratory rate 24 breaths/min, SpO_2_ (Saturation of Peripheral Oxygen) 92% on room air, blood pressure 160/100 mmHg. He was moderately anemic with bilateral moderate pitting pedal edema. On chest auscultation, bilateral basal crepitation was present. His body mass index (BMI) was 26.7 kg/m^2^ (96th percentile) (Height 4′8″, weight 119 pounds) and waist circumference was 80 cm (95th percentile).^5^ Upper limb postaxial polydactyly was present (Figure 1). There was no pubic or axillary hair. The patient also had a high arched palate and micropenis (Stretched penile length 1.5 cm) with a testicular volume of 3 mL (Tanner stage 1) (Figure 2).^6^ His intelligence quotient (IQ) was 70 (Wechsler Intelligence Scale for Children).^7^ Other systemic examinations were unremarkable.

Table 1 presents a comprehensive overview of all relevant investigation results conducted on our patient.

**TABLE 1:** Investigation results.

Test	Result	Reference
Hemoglobin	8.7 g/dL	13.5–17.5 g/dL in male
Serum creatinine	13.8 mg/dL	0.6–1.2 mg/dL
Estimated glomerular filtration rate (eGFR)	5 mL/min/1.73m^2^	90 to 120 mL/min/1.73m^2^
Blood urea	170 mg/dL	7–18 mg/dL
S. Sodium	130 mEq/L	135–145 mEq/L
S. Potassium	5.8 mEq/L	3.5–5.0 mEq/L
S. Calcium	7.1 mg/dL	8.4–10.2 mg/dL
Inorganic phosphate	11.7 mg/dL	3.0–4.5 mg/dL
Urine analysis	Albumin‐ ++, red blood cell cast	
24‐h urinary total protein	2.1 gram	<150 mg/24 h
pH	7.3	7.35–7.45
pCO2	32 mmHg	33–45 mmHg
HCO3^−^	20 mEq/L	22–28 mEq/L
ALT	143 U/L	8–20 U/L
AST	97 U/L	8–20 U/L
Total cholesterol	280 mg/dL	<200 mg/dL
Triglyceride	220 mg/dL	35–160 mg/dL
HDL	29 mg/dL	>40 mg/dL
LDL	190 mg/dL	<100 mg/dL
S. testosterone	0.8 nmol/L	10–35 nmol/L during puberty in male
LH	<0.12 IU/L	1.24–7.8 IU/L during puberty in male
FSH	<0.11 IU/L	0.3–10.0 IU/L during puberty in male
HBsAg	Negative	
Anti HCV	Negative	
S. TSH	1.15 μIU/ml	0.5–5.5 μIU/mL
RBS	5.1 mmoL/L	<7.8 mmoL/L
Electrocardiogram (ECG)	Sinus rhythm	
Echocardiogram	Normal	
Chest X‐ray	Features of pulmonary edema	

**FIGURE 3:**
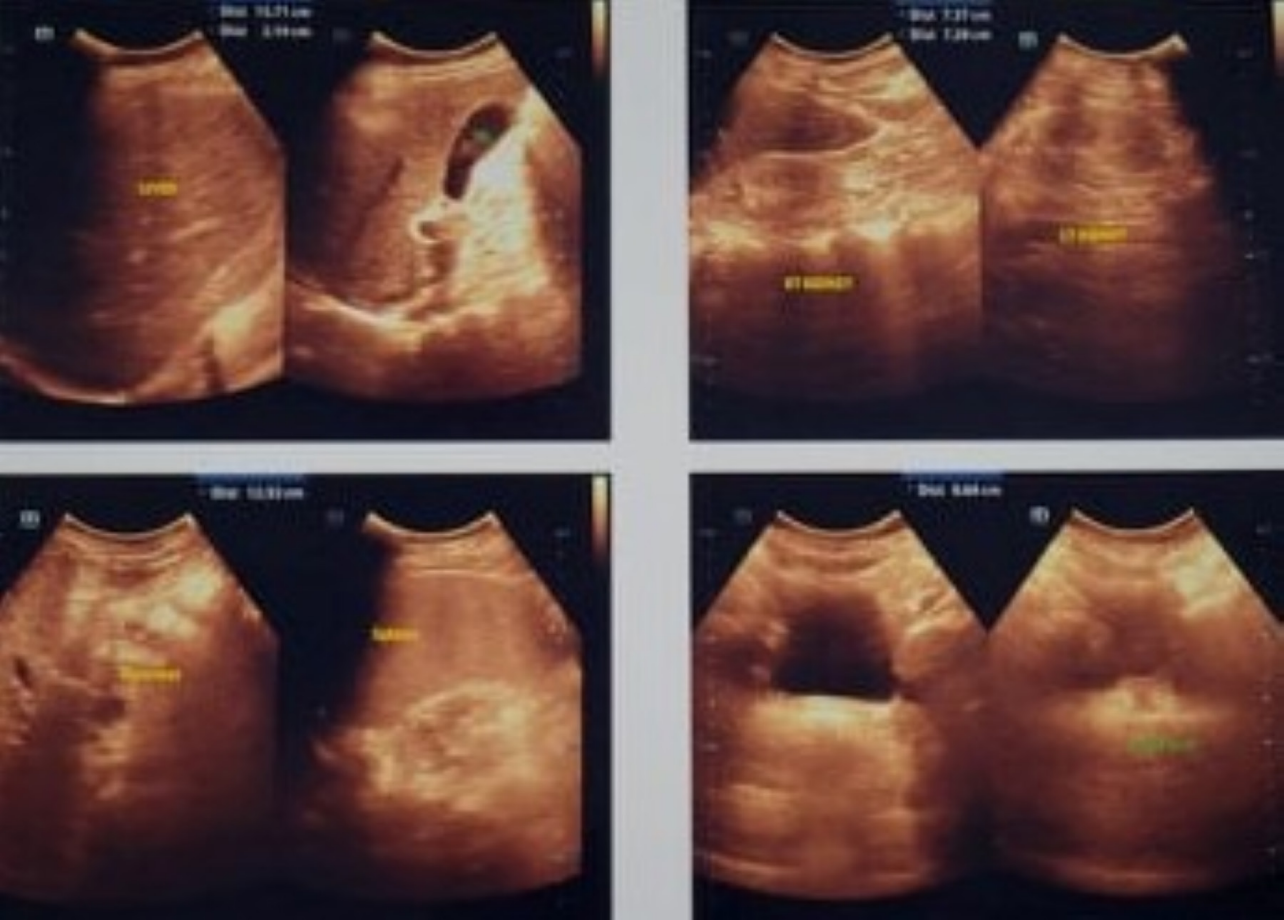
Ultrasonography of the abdomen showing bilateral renal parenchymal disease, fatty change in the liver (grade 2), and cholelithiasis.

**FIGURE 4:**
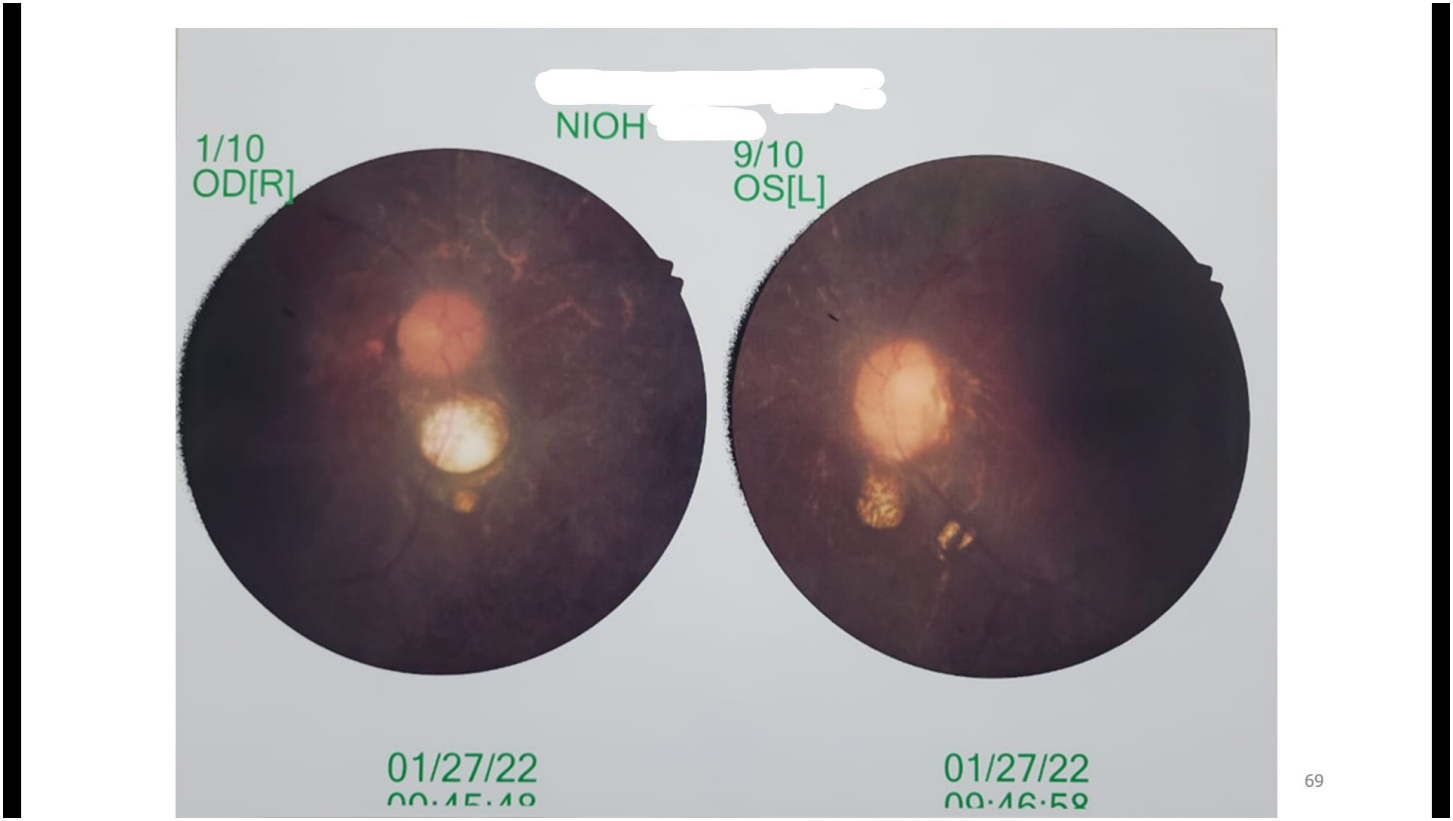
Color fundus Photograph of both eyes showing features of retinitis pigmentosa.

Investigation revealed moderate normocytic normochromic anemia, and grossly impaired renal function tests. An arterial blood gas (ABG) analysis showed mild metabolic acidosis. Liver enzymes were also elevated. Fasting lipid profile revealed dyslipidemia. Hormone analysis showed secondary hypogonadism. Ultrasonography of the whole abdomen revealed bilateral renal parenchymal disease (right kidney 8.5 cm, left kidney 8.3 cm, cortical scarring present), fatty change in the liver (grade 2), and cholelithiasis (Figure 3). Renal biopsy results were suggestive of chronic glomerulonephritis. Audiometry was reported as a mild conductive type of hearing loss in both ears. Color fundus photography revealed features of retinitis pigmentosa ( Figure 4 ). We planned for comprehensive genome sequencing, which was not currently available at our hospital.

Based on the overall clinical picture, we diagnosed the patient with BBS, as our patient had six primary features and two secondary features, as suggested by Forsythe and Beales (2013).^1^ Emergency renal replacement therapy (RRT) was initiated after consultation with the nephrology department. We also advised him to continue maintenance hemodialysis. On a 1 month follow‐up of regular hemodialysis, bilateral pedal edema, and anemia improved. Hypertension was well controlled. Serum creatinine, liver function test, and dyslipidemia improved significantly. Follow‐up ophthalmological evaluation showed mid‐peripheral scotomata with normal visual acuity and color vision. Patient education and genetic counseling regarding this condition were offered. The patient was scheduled on regular follow‐up to delay the disease complications as much as possible.

## 3 DISCUSSION

BBS was first described independently in 1920 by French Physician Georges Louis Bardet and Hungarian‐Austrian Pathologist Artur Biedl in 1922. Previously it was coupled with Laurence‐Moon Syndrome (LMS) and was referred to as Laurence–Moon–Bardet–Biedl Syndrome (LMBBS).^8^ This term is no longer in use as patients with LMS usually present with progressive spastic paraparesis but does not have polydactyly or obesity, which are the key features of BBS. It is an autosomal recessive genetic disorder. Till now, 26 genes have been identified (BBS1 to BBS22, NPHP1, CEP19, SCAPER, and SCLT1), which contribute to the development of this syndrome.^9^ Bardet–Biedl Syndrome is classified as a non‐motile ciliopathy. Out of the 26 genes responsible for BBS, 8 encode components of BBSome complex which plays an important role in primary cilia homeostasis, 3 encode Chaperonin like protein assisting BBSome formation, 3 encode components of Intra‐flagellar trafficking (IFT‐B), and other genes encode GTP‐binding protein, E3 ubiquitin ligase, Meckel syndrome type 1 protein, Centrosomal Protein 290 (CEP290), Sodium channel, and clathrin linker 1, PCP Protein Required for Ciliogenesis, regulates Sonic Hedgehog signaling, recruits the RABL2B GTPase to the ciliary base, controls cell‐matrix signaling, controls ciliary dynamics, and disassembly, etc.^9^ The most common feature found in patients with BBS is Retinal Dystrophy.^10^ Almost all patients reach legal blindness by the third decade of life.^10^ Obesity is the second most common feature in patients with BBS, which generally begins in early childhood.^11^ Limb deformities such as polydactyly, brachydactyly, prominent gap between toes, syndactyly, and fifth finger clinodactyly are also commonly seen.^12^ BBS patients develop learning difficulties, some extent of intellectual disability, and this decrease in IQ level is invariably associated with a visual difficulties.^8^ Genital abnormalities, mainly hypogonadism, are more common in males than females. BBS patients frequently develop cryptorchidism, micropenis, and short scrotum.^13^ Renal involvement leads to a poor prognosis, typically observed in the third or fourth decade of life.^14^ The exact histological pattern of renal involvement varies from patient to patient. The ones most described in various literature are chronic interstitial nephritis, Membranoproliferative glomerulonephritis (MPGN), ultrastructural changes in glomerular basement membrane, etc.^15^

As BBS has no definitive treatment, a supportive and symptomatic approach is needed while treating such patients.^9^ These include training blind patients, rehabilitating intellectually disabled patients, hearing aids, speech therapy, exercise for weight management, and surgery for polydactyly.^9^ Some studies suggest adopting a low‐protein & low‐calorie diet may help in both obesity control and halting the progression of renal impairment.^16^ Regular screening should also be performed for early detection of Insulin resistance, metabolic syndrome, hypertension, and renal impairment.^17^ When renal failure occurs later in life, RRT (e.g., hemodialysis, peritoneal dialysis, and even kidney transplantation) may be required.

In Table 2, the primary and secondary characteristics of BBS in comparison to our patient have been described. For a patient to be clinically classified as BBS, four primary features or three primary and two secondary features must be present.^1^ Our patient had six primary and two secondary features.

**TABLE 2:** Primary and secondary characteristics of BBS in our patient.

Modified diagnostic criteria of BBS		Our patient
Primary features	Rod‐cone dystrophy (93%)	+
	Polydactyly (63%–81%)	+
	Obesity (72%–92%)	+
	Learning disabilities (61%)	+
	Genital anomalies (59%–98%)	+
	Renal anomalies (53%)	+
Secondary features	Speech delay (54%–81%)	+
	Strabismus/cataracts/astigmatism (6%–90%)	−
	Brachydactyly/ syndactyly (8%–95%)	−
	Developmental delay (50%–91%)	+
	Ataxia/poor coordination/imbalance (40%–86%)	−
	Mild spasticity (especially lower limbs)	−
	Diabetes mellitus (6%–48%)	−
	Dental anomalies (51%)	−
	Congenital heart disease (7%)	−
	Anosmia/hyposmia (60%)	−

Our patient was presented to us with acute kidney injury on top of chronic kidney disease, however without a previous diagnosis of BBS. Laboratory findings of our patient revealed non‐nephrotic range proteinuria, which may be attributable to his renal biopsy findings of chronic glomerulonephritis. However, Renal imaging did not show any pathological abnormalities other than poor corticomedullary differentiation and cortical scarring. Ophthalmological evaluation of our patient showed early features of retinitis pigmentosa, but he did not have any visual problems. Although our patient was a classic case of BBS and fulfilled most of the diagnostic criteria, he remained undiagnosed until the age of 14 years. This delay in diagnosis made him vulnerable to multiple complications. The lack of suspicion of a rare disease by the patient's parents and primary medical professionals was one of the significant causes contributing to his prolonged undiagnosed period. Consanguineous marriage is considered a significant risk factor for BBS.^18^ However, our patient did not provide a history of the parents' consanguineous marriage. Genetic testing to confirm our clinical diagnosis of BBS could not be done due to its unavailability in Bangladesh.

## 4 CONCLUSION

Patients with the characteristic phenotype of central obesity, polydactyly and underdeveloped external genitalia should be evaluated for BBS. High clinical suspicion of this rare disease is the key. Early diagnosis will prevent or delay morbidity and mortality resulting from the delay in diagnosis. Renal function tests and ophthalmological evaluation should be done regularly to detect early complications.

## AUTHOR CONTRIBUTIONS

**Mohammad Rasel:** Writing – original draft; writing – review and editing. **Ashif Istiak:** Writing – original draft. **Afra Saiara:** Writing – original draft. **Abdullah Al‐Jubair:** Writing – review and editing. **Shariful Matin:** Writing – review and editing. **Gobinda Chandra Roy:** Supervision.

## FUNDING INFORMATION

The authors received no financial support for authorship, and/or publication of this article.

## CONFLICT OF INTEREST STATEMENT

All the authors of this manuscript have no conflict of interest.

## ETHICS STATEMENT

Ethics approval is not required for de‐identified single case reports based on institutional policies.

## CONSENT STATEMENT

Written informed consent was obtained from the patient's father to publish this case report in accordance with the journal's patient consent policy. A copy of the written consent is available for review by the editor in chief of this journal on request.

## Data Availability

Data sharing is not applicable to this article as no datasets were generated or analyzed during the current study.
